# N-Cadherin Dynamically Regulates Schwannoma Migration and Represents a Novel Therapeutic Target in NF2-Related Schwannomatosis

**DOI:** 10.21203/rs.3.rs-8817797/v1

**Published:** 2026-02-19

**Authors:** Han T.N. Nguyen, Melanie Fisher, Ruiqi Zhou, Taha A. Jan, Yin Ren

**Affiliations:** 1 Department of Otolaryngology - Head and Neck Surgery, The Ohio State University Wexner Medical Center, Columbus, OH; 2 Departments of Otolaryngology – Head and Neck Surgery, Cell and Developmental Biology, and Hearing and Speech Sciences, Vanderbilt University Medical Center, Nashville, TN

## Abstract

NF2-related schwannomatosis (NF2-SWN) is a devastating tumor predisposition syndrome marked by multiple schwannomas and substantial morbidity. Vestibular Schwannoma (VS), the hallmark tumor, causes deafness, vertigo and potentially fatal brainstem compression. A subset develops brainstem adhesions, making surgery - the only treatment option – highly risky in the absence of FDA-approved therapies. During NF2-SWN progression, schwannoma cells migrate from the extracellular matrix (ECM)-rich internal auditory canal to the arachnoid-lined brainstem, yet mechanisms driving this transition remain incompletely defined. We previously demonstrated that adherent schwannoma is associated with elevated matrix metalloproteinase-9 (MMP-9) activity. N-cadherin (N-cad), a key cell-matrix adhesion molecule and regulator of cancer cell migration, has not been studied in NF2-SWN. Integrating RNA sequencing, multiple NF2 schwannoma mouse models and primary human VS cultures, we demonstrate that N-cad is overexpressed in sporadic and NF2-associated VS. N-cad differentially regulates VS spheroid migration – promoting motility on astrocytes but restraining on ECM. MMP-9 cleaves N-cad to drive schwannoma proliferation through IL-6/STAT3 and NF-κB signaling. Pharmacologic and genetic inhibition of N-cad synergized with dasatinib and brigatinib, two kinase inhibitors with efficacy in NF2-SWN, to suppress schwannoma proliferation and tumor growth. These findings establish N-cad as a central regulator of schwannoma migration and a novel therapeutic target.

## Introduction

NF2-related schwannomatosis (NF2-SWN), also known as neurofibromatosis type 2) is a devastating autosomal dominant tumor disposition syndrome characterized by the development of multiple schwannomas throughout the nervous system, with bilateral vestibular schwannomas (VSs) as the defining hallmark [1]. In addition to NF2-associated VS, sporadic VS represents the fourth most common primary intracranial neoplasm with an estimated prevalence of 1 in 2,000 adults and 1 in 500 adults over 70-years-old [2]. A subset of schwannomas exhibits aggressive behavior characterized by rapid growth, development of dense adhesions to the brainstem and cranial nerves, resulting in marked neurological morbidities including deafness, vertigo, hydrocephalus, brainstem compression and even death [3, 4].

There are currently no FDA-approved pharmacotherapies for sporadic or NF2-associated schwannoma. Off-label use of chemotherapies such as bevacizumab and brigatinib provides limited efficacy and significant toxicity [5–8]. Radiation is ineffective for large or rapidly growing NF2 schwannomas and carries a small but fatal risk of malignant transformation. Surgical resection remains the standard of care despite its invasive nature. Most NF2-SWN patients endure multiple operations due to relentless tumor growth, with the risk of neurological injury increasing with each operation. Furthermore, surgical morbidities are significantly elevated in adherent schwannomas [9, 10], where disrupted arachnoid planes increases the likelihood of incomplete tumor removal and damage to cranial nerves, brain parenchyma and brainstem [11].

NF2-SWN progression is a coordinated process driven by both tumor growth and migration, influenced by dynamic crosstalk between schwannoma cells, stromal cells, and the extracellular matrix (ECM). As schwannoma cells transition from the ECM-rich, cellularly sparse internal auditory canal (IAC) to the cerebellopontine angle (CPA), they migrate collectively along neuronal fibers, vessels, and perineural ECM [12–15]. These movement trajectories, regulated by cadherin- and integrin-mediated cell-cell and cell-matrix adhesions, mirror Schwann cells (SC) migration during development and after nerve injury, where SCs traverse damaged axons to create a regenerative milieu [16]. On a molecular level, homotypic adhesions promote collective SC movement, while heterotypic adhesions with astrocytes and glia facilitate brain extension and adherence to cranial nerves [17–20]. Recent single-cell RNA sequencing (scRNA-seq) studies revealed that VS-associated SCs adopt a “repair-like” and MHC-II antigen-presenting “injury-like” state to recruit macrophages and promote tumor growth, suggesting that VS co-opts regenerative SC migration pathways to drive progression [21, 22].

N-cadherin (N-cad), a regulator of cell–cell adhesion, plays crucial roles in cancer hallmarks including invasion and epithelial-mesenchymal transition [23–29]. N-cad maintains neuroepithelial integrity and guides neural progenitor and neural crest cell migration [30, 31]. As a transmembrane receptor, N-cad facilitates homophilic cell adhesion and contact-dependent signaling that modulate cell cohesion and cytoskeletal remodeling [32–35]. N-cad also interacts with fibroblast growth factor receptor and integrins to regulate MAPK and phosphatidylinositol 3-kinase (PI3K)-AKT signaling and adhesion plasticity – processes critical for both neural development and tumorigenesis [36, 37]. N-cad is linked to the actin cytoskeleton via catenin, enabling SC migration and turnover of adhesions during nerve repair [38]. Studies in glioma show that tumor cells hijack these programs to adapt to diverse extracellular environments and enhance invasion, with elevated N-cad expression associated with poor prognosis [39]. However, the mechanisms by which N-cad shapes schwannoma progression remain elusive.

Prior studies have shown that tumor microenvironment (TME) alterations play a key role in schwannoma progression. N-cad shapes how schwannoma cells interact with TME by regulating adhesion to stromal/ECM cues and activating pro-survival signaling [40, 41]. Here, utilizing schwannoma cell lines, a novel 3-dimensional (3D) tumor spheroid model, primary human VS cultures established from surgical specimens, and multiple mouse schwannoma models, we investigate the role of N-cad in schwannoma migration and progression. N-cad promoted NF2 schwannoma cell migration across astrocytes but restricted movement on ECM, with distinct localization patterns observed during collective migration of primary VS. N-cad activated IL-6/STAT3 and NF-κB signaling, while N-cad knockdown reduced cancer stemness marker expression. Importantly, N-cad depletion sensitized NF2 schwannoma to dasatinib (a broad Src-family TKI) and brigatinib (a multi-kinase inhibitor) - both used in Phase II clinical trials for NF2-SWN patients with progressive tumors – by synergistically inhibiting focal adhesion kinase (FAK), phosphorylated Src family kinase (Src) and PI3K/Akt. Together, these results establish N-cad as a driver of migratory and growth programs in schwannoma and a promising therapeutic target for NF2-SWN.

## Methodology

### Cell culture

Mouse *Nf2*^−/−^ schwannoma cells (MD-MSC, gift from Lei Xu, Massachusetts General Hospital) were derived from embryonic Schwann cells isolated from Nf2^loxP/loxP^ mice at embryonic day 13.5 (E13.5). Cells were infected with a Cre recombinase-expressing adenovirus to excise exon 2 of the Nf2 locus, generating a functional merlin-deficient (*Nf2*^−/−^) Schwann cell line. NF2-mutant human schwannoma cells (HEI-193), were cultured in standard DMEM-based media supplemented with 10% fetal bovine serum (FBS, Gibco), 1% penicillin/streptomycin, and 10 μM forskolin. Details on primary VS culture are described previously [42, 43] and were established from tumor specimens obtained under IRB approval from the Ohio State University (IRB#1994H0241).

### Lentiviral shRNA and siRNA N-cadherin modulation

Stable N-cad knockdown was achieved by transducing cells with lentiviral particles encoding a *CDH2*-targeting shRNA (Sigma-Aldrich MISSION TRC TRCN0000053978) in the pLKO.1-puro-CMV-tGFP backbone; a non-targeting shRNA lentivirus served as control. For siRNA knockdown, MD-MSC cells were transfected with mouse *Cdh2* siRNA (Thermo Fisher; Silencer^™^ ID 160128, AM16708) or a non-targeting siRNA control using Lipofectamine RNAiMAX (Invitrogen/Thermo Fisher; 13778030) according to the manufacturer’s protocol.

### Animal models

Athymic female nude mice (6–8 week old, Jackson Labs, Bar Harbor, ME, strain code: 007850, homozygous) and NSG mice (NOD.Cg-*Prkdc*^*scid*^ *Il2rg*^*tm1Wjl*^/SzJ; 6–8 week old, Jackson Labs, Bar Harbor, ME, strain code:005557) were obtained under the protocol approved by Institutional Animal Care and Use Committee (Protocol#: 2022A00000043). A NF2 schwannoma allograft model was established by injecting MD-MSC cells subcutaneously in the flanks of athymic nude mice (5,000 cells per mouse). Mice were randomly assigned to vehicle control, Dasatinib (MedchemExpress, HY-10181) combine with brigatinib (MedchemExpress, HY-12757) (D+B), or D+B plus the N-cad antagonist ADH-1 (MedchemExpress, HY-13541). A second xenograft model was established by injecting HEI-193 cells expressing shRNA targeting *N-cad* or a control shRNA into the subcutaneous flanks of athymic nude mice (10×10^6^ cells per mouse). A third xenograft schwannoma model was established by injecting HEI-193 cells expressing sh*Ncad* or sh*Ctrl* subcutaneously into the flanks of NSG mice (10×10^6^ cells per mouse).

### Transcriptomic and single-cell analyses

Public microarray datasets (GSE141801, GSE108524, GSE39645) and a published scRNA-seq dataset (GSE216784) were re-analyzed to compare VS with control vestibular nerves [21]. After log_2_ normalization and sample annotation, differentially expressed adhesion-related genes, including N-cad family members and integrins were identified and visualized. scRNA-seq data from Barrett et al. (2023) was downloaded from NCBI’s Gene Expression Omnibus. scRNA-seq data were obtained as preprocessed count matrices. Initial quality control assessment confirmed that standard quality control had already been applied to the dataset; therefore, all cells from the original data were retained for downstream analyses. To minimize batch effects of input samples, we chose to only include fresh tumor tissue samples (n=11), including SCH1–6, SCH9, SCH13, and SCH14, yielding a total of 63,931 cells. All analyses were performed in Python using scanpy (v1.9.8). For downstream processing, we normalized the count matrix to total library size and applied a log normalization. We then selected the top 3,000 highly variable genes to perform PCA. Batch effects across the nine runs were corrected using the BBKNN algorithm [44], where the neighborhood graph was constructed based on the top 50 principal components. This integrated graph served as the input for UMAP visualization and Leiden clustering. Final cell type annotations were assigned by mapping each cell to its corresponding label defined in the original study’s metadata file, including Schwann cell subtypes (nmSC and myeSC) and stromal/immune populations. CDH2 expression was plotted as log2 normalized expression on the reduced dimensions UMAP plot. Differential expression was performed within Schwann cell subclusters, and cadherin-related DEGs were identified by intersecting subcluster DEGs with a curated cadherin gene list; representative genes were plotted in GraphPad Prism (v10.3).

### Drug synergy analysis

Schwannoma cells were treated with brigatinib, dasatinib and pictilisib alone or in combination. Cells were exposed to a matrix of drug concentrations determined from individual IC_50_ for 72 hours. Single agent dose response curves and IC_50_ were fitted using a four-parameter logistic model. Cell viability was measured using a commercial bioluminescent ATP assay (CellTiter-Glo, Promega). Synergy scores were generated with Combenefit (Cancer research UK) to visualize areas of greater than additive inhibition. A synergistic interaction was defined by an excess over Loew or HSA expectation (synergy score > 10 or combination < 1).

### Code and Data Availability

All transcriptomic data were obtained from publicly available NCBI GEO datasets, including microarray and scRNAseq data. These include microarray datasets (GSE141801, GSE108524, GSE39645), and a scRNA-seq dataset (GSE216784). Data analysis utilized standard Python code and has also been deposited in a publicly available GitHub repository, including code to generate figures (https://github.com/tahajanlab/published).

## Results

### 1. N-cad is highly expressed in human schwannomas

**Figure 1.**
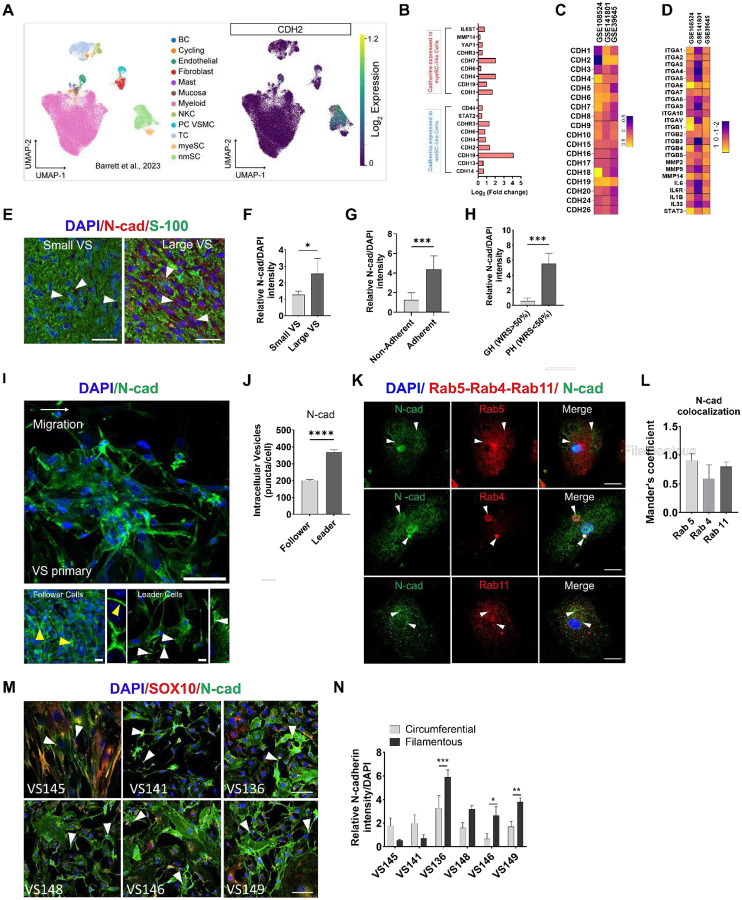
N-cad expression and localization correlate with tumor size, migration dynamics, and hearing in human schwannoma (A) Single-cell RNA sequencing (scRNA-seq) UMAP visualization of schwannoma cell subpopulations (left) showing CDH2 enrichment within Schwann cell clusters (right) (B) Bulk transcriptomic profiling of human schwannoma in non-myelinating Schwann cells (nmSC, blue) and myelinating Schwann cells (myeSC, red) showing enrichment of cadherin family genes including CDH2 (N-cadherin) in nmSC-like cells. (C-D) Heatmaps summarizing expression of cadherin and integrin adhesion gene families in schwannomas. (E-F) Representative immunofluorescence staining and quantification of N-cad expression in NF2-SWN tumors of different size (small [n=4] = average diameter 1.1 cm; large [n=4] = average diameter 3.9 cm). N-cad (red), S-100 (green), DAPI nuclear counterstain (blue). Scale bar, 50 μm. (Bars represent mean ± SD; *P < 0.05). (G) Quantification of N-cad mean fluorescence intensity in human schwannoma tissues comparing adherent to non-adherent schwannomas (n= 8; Bars represent mean ± SD, **P < 0.01). (H) Quantification of N-cad mean fluorescence intensity in NF2-SWN patients with good hearing (GH, defined as having word recognition score [WRS] > 50%) and poor hearing (PH, defined as having, WRS < 50%), (n=8; Bars represent mean ± SD, ***P < 0.001). (I) N-cad expression in primary VS culture showing differential localization in migrating leader versus follower cells. Insets show representative cells from each group. Yellow arrowheads indicate cell-cell junctions in follower cells and white arrowheads indicate vesicles in leader cells. Scale bar 50 μm or 10 μm (inset). (J) Quantification of intracellular N-cadherin vesicles (puncta/cell) in leader and follower cells (n=15; Bars represent mean ± SD, ****P < 0.0001). (K) Representative immunofluorescence images showing colocalization between N-cad and endosome markers. Arrow heads indicate N-cad positive endocytic vesicles colocalized with Rab5-expressing early endosomes and Rab4-/Rab11-positive recycling endosomes. Scale bar, 10 μm. (L) Mander’s colocalization coefficients of (K) (n=15–20 cells examined per endosome marker). (M) Immunofluorescence of primary VS cultures from six patients co-stained for N-cad (green) and SOX10 (red). Scale bar, 25 μm. (N) Quantification of circumferential and filamentous N-cad signal intensity across patient-derived primary VS cell lines (n=10; Bars represent mean ± SD, *P < 0.05, **P < 0.01, ***P < 0.001).

N-cad-mediated adhesion preserves tissue architecture while enhances cancer invasion through dynamic cell-ECM interactions and pro-survival signaling during EMT [39, 45]. To determine the cellular source of N-cad in NF2-SWN schwannoma cells, we analyzed published single cell RNA (scRNA)-seq data comprised of 15 fresh sporadic schwannomas. *CDH2* (N-cad) was markedly enriched in a non-myelinating, injury-like Schwann cell (nmSC) subpopulation with minimal expression in endothelial or immune cells (Fig. 1A–B). Analysis of 3 published microarray datasets (GSE108524, GSE141801, GSE39645) confirmed *CDH2* is highly overexpressed in human schwannomas, accompanied by upregulation of matrix metalloproteinases (*MMP-2/9/14*) and integrins (*ITGA6*, *ITGB1*, *ITGAV*) (Fig. 1C–D), consistent with activation of an adhesion-ECM remodeling transcriptional program.

We next evaluated the spatial localization of N-cad in human schwannoma tissues. Immunofluorescence demonstrated elevated N-cad level per tumor area in large compared to small NF2-SWN tumors (average diameter 3.9 vs. 1.1 cm, p=0.021, Fig. 1E–F). N-cad expression was elevated in aggressive NF2-SWN adherent to the facial nerve and brainstem and in tumors from patients with poor hearing (word recognition score, [WRS]<50%), linking increased N-cad to adverse tumor phenotypes (Fig. 1G–H). Confocal imaging of migrating primary VS cultures demonstrated distinct leader-follower organization, with leader cells exhibiting neurite-like, filamentous cell-cell contact and perinuclear N-cad vesicles, whereas follower cells exhibiting circumferential, epithelial-like contacts (Fig. 1I–J).

Given the role of endocytic trafficking in regulating surface N-cad levels during cell migration, we next assessed the localization of N-cad puncta relative to endosomal compartments. N-cad partially colocalized with Rab5-expressing early endosomes and Rab4-/Rab11-positive recycling endosomes (Fig. 1K–L). Leader cells showed preferential N-cad endocytosis toward migration fronts, but the magnitude of this response varied across patient-derived primary cultures, likely due to inter-patient heterogeneity in N-cad dynamics (Fig. 1M–N). Together, these data identify injury-like nmSCs as a major source of N-cad in NF2-SWN and link elevated N-cad expression and recycling to aggressive tumor phenotypes and collective migration.

### 2. N-cad suppresses NF2-SWN schwannoma cells migration on ECM but enhances migration on astrocytes

**Figure 2.**
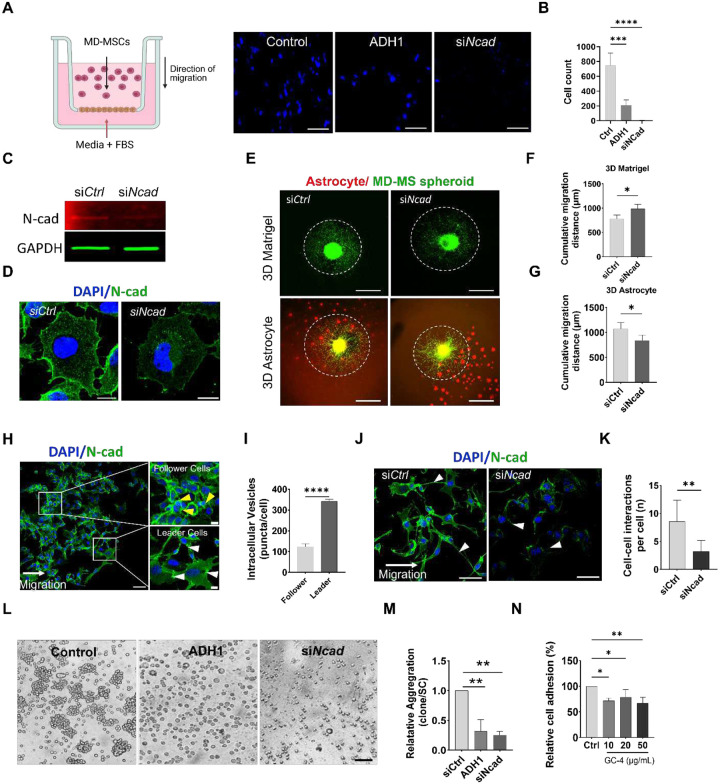
N-cad controls context-dependent migration and cell–cell cohesion. (A) Schematic of the transwell migration assay with representative fluorescence images of migration of MD-MSC cells (DAPI) treated with ADH-1 (1 mg/mL), or si*Ncad*. Scale bar, 100 μm. (B) Quantification of migrated cell counts from (A) (n=12; Bars represent mean ± SD, ***P < 0.01; ***P < 0.001). (C) Immunoblot confirming N-cad knockdown in MD-MSC cells treated with si*Ncad* vs si*Ctrl* cells. GAPDH serves as the loading control. (D) Representative immunofluorescence images showing reduced N-cad (green) expression following siRNA treatment. Scale bar, 10 μm. (E) Representative images of spheroid outgrowth/migration in 3D Matrigel (top) or in 3D astrocyte co-culture (bottom) comparing si*Ctrl* vs si*Ncad* cells; dashed outlines indicate spheroid boundaries. Scale bar, 200 μm. (F–G) Quantification of cumulative migration distance in 3D Matrigel (F) and 3D astrocyte conditions (G) (n=10; Bars represent mean ± SD, *P < 0.05). (H) Representative immunofluorescence images of migrating cultures showing leader–follower organization; boxed regions highlight leader and follower cells. N-cad is pseudocolored in green, yellow arrowheads indicate cell-cell junctions in follower cells and white arrowheads indicates vesicles in leader cells. Scale bar 50 μm or 20 μm (inset). (I) Quantification of intracellular N-cadherin puncta/vesicles per cell in leader vs follower populations. (n=15; Bars represent mean ± SD, *P < 0.05). (J) Representative migration fields for si*Ctrl* vs si*Ncad* cells; arrowheads indicate cell–cell contacts during collective movement. Scale bar, 20 μm. (K) Quantification of cell–cell interactions per cell for (J) (n=10; Bars represent mean ± SD, *P < 0.05). (L) Representative brightfield images of aggregation assay of MD-MSC cells treated with ADH-1 (1 mg/mL), or si*Ncad*. Scale bar, 100 μm. (M) Quantification of relative cell aggregation from (L) (n= 8; Bars represent mean ± SD, **P < 0.01. (N) Quantification of MD-MSC cell adhesion following treatment with GCA at indicated doses. (n=10; Bars represent mean ± SD, *P < 0.05, **P < 0.01).

To investigate how N-cad regulates schwannoma cell motility, we performed a 2D transwell assay using merlin-deficient mouse schwannoma cells (MD-MSC) and NF2-mutant human schwannoma cells (HEI-193). Pharmacologic inhibition of N-cad using ADH-1, a cyclic peptide antagonist targeting the HAV-binding domain, significantly reduced MD-MSC cell spheroid migration (Fig. 2A–B, **Fig. S1A-B**). Short-interfering RNA (siRNA) and lentiviral shRNA knockdown of N-cad in MD-MSC and HEI-193 cells significantly reduced N-cad mRNA and protein expression (Fig. 2C–D, **Fig. S1C-E**). Notably, MMP-9 expression was also reduced, suggesting that N-cad depletion coincides with attenuation of ECM remodeling (**Fig. S1E**). To more accurately model the schwannomas microenvironment, we generated a 3D schwannoma spheroid model using either synthetic ECM (Matrigel) or in co-culture with astrocytes to mimic neural interactions. N-cad knockdown significantly increased schwannomas migration on Matrigel/laminin-rich matrix but reduced their migration on astrocytes (Fig. 2E–G, **Fig. S1F-H**). Similar findings were observed in HEI-193 spheroids (**Fig. S1I-K**). Thus, N-cad regulates schwannoma migration in a context-dependent manner, promoting migration in astrocyte-enriched conditions while restricting migration on ECM.

Given that cadherin surface levels are dynamically regulated through endocytosis, recycling and degradation, we examined the membrane trafficking dynamics of N-cad in migrating schwannoma monolayers. Wound scratch assay revealed filamentous N-cad junctions at the migration leading edge and circumferential, epithelial-like junctions in follower cells (Fig. 2H–I), resembling tumor microtube-like structures [15, 46]. Treatment with si*Ncad* reduced the cell-cell connection at the migration front (Fig. 2J–K). and pharmacologic N-cad blockade using ADH-1 or the neutralizing antibody GC-4 [47] reduced cell–cell adhesion and aggregate formation (Fig. 2L–N; **Fig. S1L-N**). These trafficking features indicate that leader cells internalize and recycle N-cad to sustain cohesive, front-directed protrusion in migrating schwannoma clusters.

### 3. N-cad activates IL-6/STAT3 and NF-kB signaling

**Figure 3.**
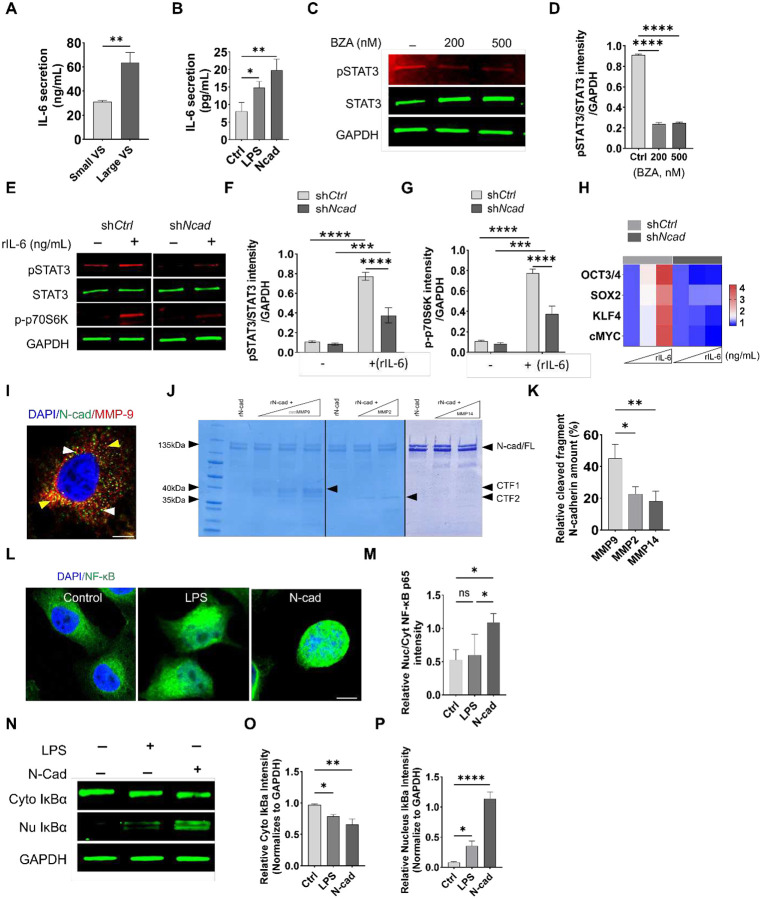
N-cad promotes IL-6/STAT3–mTOR signaling and NF-κB activation and is susceptible to MMP-mediated cleavage. (A) IL-6 secretion measured by ELISA in conditioned media from primary VS cultures from small (N=4, average diameter 1.1 cm) and large (N=5 average diameter 3.9 cm) NF2-SWN tumors. (Bars represent mean ± SD, **P < 0.01). (B) IL-6 secretion from HEI-193 cells following stimulation with lipopolysaccharide (LPS) or recombinant N-cad compared with control. (Bars represent mean ± SD, *P < 0.05, **P < 0.01). (C) Immunoblot of phosphorylated STAT3 (pSTAT3), total STAT3, and GAPDH in cells treated with bazedoxifene (BZA; 200 or 500 nM). (D) Densitometric quantification of pSTAT3 normalized to total STAT3 from (C) (Bars represent mean ± SD, ****P < 0.0001. (E) Immunoblot of pSTAT3, total STAT3, phosphorylated p70S6K (p-p70S6K), and GAPDH in HEI-193 cells expressing either sh*Ctrl* or sh*Ncad* under basal conditions (−) or stimulated with recombinant IL-6 (rIL-6, 100 ng/mL). (F, G) Quantification of pSTAT3/STAT3 (F) and p-p70S6K (G) from (E), comparing sh*Ctrl* and sh*Ncad* cells with or without rIL-6 treatment (Bars represent mean ± SD, ***P < 0.001, ****P < 0.0001). (H) Heatmap showing relative expression of stemness-associated factors (OCT3/4, SOX2, KLF4, cMYC) in sh*Ctrl* and sh*Ncad* cells across increasing doses of rIL-6. (I) Representative immunofluorescence images showing N-cad (green) and MMP-9 (red) with nuclear counterstain (DAPI; blue); yellow arrowheads indicate MMP-9, white arrowheads indicate N-cad. Scale bar, 10 μm. (J) In vitro digestion of N-cad by recombinant MMP-9, MMP-2, or MMP-14 showing generation of N-cad fragments; arrowheads indicate cleavage products, including C-terminal fragments (CTF1/CTF2) relative to full-length (FL) N-cadherin. (K) Quantification of cleaved N-cadherin fragments relative to FL (n=3; Bars represent mean ± SD, *P < 0.05, **P < 0.01). (L) Representative immunofluorescence images showing intracellular co-localization of NF-κB after LPS or N-cad stimulation. Scale bar, 10 μm. (M) Quantification of NF-κB p65 nuclear-to-cytoplasmic intensity ratio (Bars represent mean ± SD, ns, *P < 0.05). (N) Immunoblot of cytoplasmic (Cyto) and nuclear (Nu) IκBα following LPS or N-cad stimulation; GAPDH served as loading control. (O-P) Densitometric quantification of cytoplasmic (O) and nuclear (P) IκBα normalized to GAPDH.

IL-6/STAT3 signaling is a key driver of NF2-SWN survival [48, 49]. To investigate whether N-cad influences IL-6/STAT3 activity, we assessed IL-6 secretion and STAT3 activation as a function of N-cad expression. Primary VS cultures from larger tumors secreted higher levels of IL-6 than those from smaller lesions (Fig. 3A). In HEI-193 cells, recombinant N-cad induced IL-6 production, though less potently than lipopolysaccharide [50] (Fig. 3B). Disruption of IL-6/GP130 signaling with bazedoxifene (BZA) suppressed STAT3 phosphorylation (Fig. 3C–D). Similarly, sh*Ncad* treatment markedly reduced STAT3 (Tyr705) phosphorylation and activation of its downstream effector kinase, p70S6K, indicating that N-cad is required to sustain IL-6/STAT3 signaling (Fig. 3E–G). Further, N-cad silencing blunted IL-6-induced upregulation of pluripotency-associated transcription factors linked to cancer stemness, including OCT3/4, SOX2, KLF4, and c-MYC [51, 52] (Fig. 3H). Together, these data identify an N-cad dependent, autocrine IL-6/STAT3 axis in schwannoma that promotes cellular proliferation and stem-like phenotypes.

Given the established crosstalk between STAT3 and NF-κB in tumor-promoting inflammatory signaling, we next investigated whether N-cad also activates NF-κB via extracellular processing [53]. Elevated MMP-9 is associated with NF2-SWN progression, and protease-mediated N-cad shedding can release bioactive ectodomains that are implicated neurite migration and outgrowth. In parallel, N-cad signaling in microglia, neurons and fibroblast has been linked to pro-survival and inflammatory programs [10, 25, 54]. In HEI-193 cells, there was an abundance of punctate N-cad and MMP-9 throughout the cytoplasm with prominent colocalization in perinuclear vesicles (Fig. 3I). Recombinant MMP-9 cleaved N-cad into discrete fragments (Fig. 3J–K). HEI-193 treated with N-cad ectodomain (rNcad) robustly activated NF-κB, as evidenced by RelA (p65) nuclear translocation and IκBα destabilization with cytosol-to-nucleus redistribution (Fig. 3L–M). Treatment with rNcad reduced cytosolic and markedly increased nuclear IkBα compared to LPS and control (Fig. 3N–P). Collectively, these results indicate that MMP-9-dependent N-cad shedding produces an active ectodomain that amplifies NF-κB signaling in NF2-SWN, positioning N-cad as a link between IL-6/STAT3 and NF-κB inflammatory signaling cascades.

### 4. N-cad depletion sensitizes schwannoma cells to treatment by dasatinib and brigatinib

**Figure 4.**
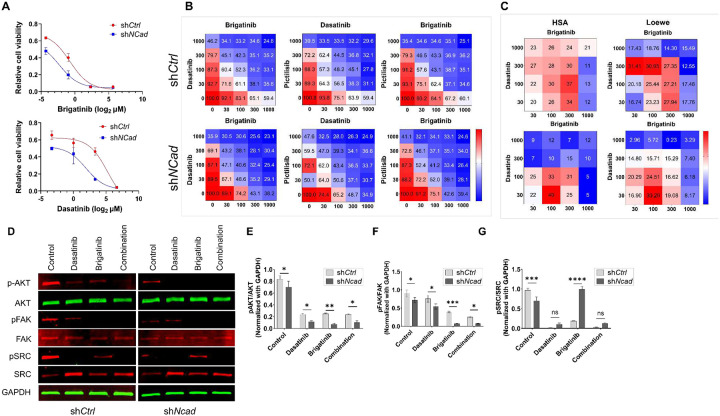
N-cad depletion enhances sensitizes NF2 schwannoma cells to kinase inhibitors and combination therapy. (A) Dose–response curves for brigatinib (top) and dasatinib (bottom) in HEI-193 cells expressing sh*Ctrl (red*) or sh*Ncad (blue*); viability is shown relative to vehicle control. (B) Drug–drug response matrices for brigatinib + dasatinib, dasatinib + pictilisib, and brigatinib + pictilisib across the indicated concentration ranges in sh*Ctrl* (top) and sh*Ncad* (bottom) cells. (C) Synergy landscapes for brigatinib and dasatinib calculated using highest single agent (HSA) and Loewe additivity models for both sh*Ctrl* and sh*Ncad* cells (positive scores indicate strongest synergy). (D) Immunoblot analysis of p-AKT, total AKT, p-FAK, total FAK, p-SRC, total SRC, and GAPDH in sh*Ctrl* (left) and sh*Ncad* (right) cells treated with vehicle (control), dasatinib (15 mg/kg), brigatinib (25 mg/kg), or both drugs in combination. (E - G) Densitometric quantification of p-AKT normalized to AKT (E), p-FAK normalized to FAK (F), and p-SRC normalized to SRC (G), all normalized to GAPDH for the indicated treatment groups in sh*Ctrl* and sh*Ncad* cells (Bars represent mean ± SD, ns, *P < 0.05, **P < 0.01, ***P < 0.001, ****P < 0.0001).

There are no FDA-approved pharmacotherapies for NF2-SWN. Targeted therapies, including the multi-kinase inhibitor brigatinib and the Src/Abl inhibitor dasatinib, have shown limited and variable efficacy in Phase I/II clinical trials. Furthermore, clinical benefit is hindered by drug resistance, non-durable responses and dose-limiting toxicities, and compensatory survival signaling, indicating that kinase inhibitors alone are unlikely to be curative [7, 55, 56]. To assess whether N-cad depletion enhances kinase inhibitor efficacy in schwannomas, we examined drug sensitivities following N-cad knockdown. Consistent with prior studies demonstrating brigatinib and dasatinib were efficacious in NF2-mutant schwannomas [57], N-cad depletion amplified this synergistic interaction, reducing brigatinib EC_50_ from 600 nM to 190 nM and dasatinib EC_50_ from 1580 nM to 570 nM (Fig. 4A). HEI-193 cells harboring sh*NCad* exhibited increased sensitivity to brigatinib and dasatinib, lowering IC_50_ values by over 55% and 80%, respectively, compared to sh*Ctrl* cells (Fig. 4B). Synergy analysis confirmed elevated Loewe excess and highest single agent (HSA) synergy scores (Fig. 4C). Mechanistically, N-cad knockdown shifted survival signaling toward ECM-dependent focal adhesion pathways. Combined brigatinib and dasatinib reduced phosphorylated focal adhesion kinase (pFAK), phosphorylated Src family kinase (pSRC), and phosphorylated protein kinase B/AKT (pAKT) in sh*NCad* cells, while ERK1/2 remained unchanged (Fig. 4D–E; **Fig S2A-B**), indicating that combination kinase and N-cad inhibition suppressed adhesion-mediated FAK/Src-PI3K/AKT axis rather than broadly shutting down mitogenic signaling. Consistent with increased reliance on the FAK/Src–PI3K/AKT axis, sh*NCad* cells were more vulnerable to pictilisib, a PI3K inhibitor (**Fig. S2C-D**). Collectively, these results indicate that N-cad loss rewires schwannoma signaling towards FAK and PI3K/AKT-dependent survival.

### 5. N-cad inhibition enhances kinase inhibitor sensitivity in human primary VS

**Figure 5.**
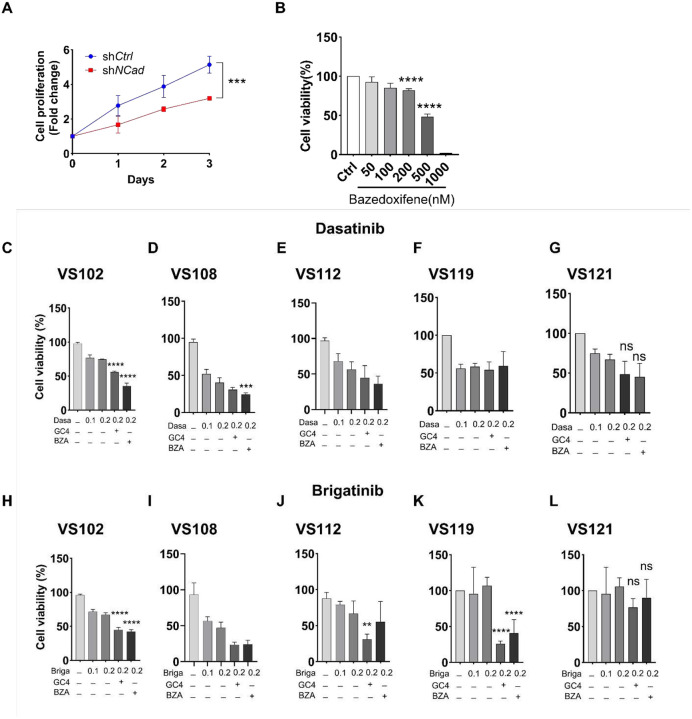
N-cad knockdown suppresses NF2 schwannoma cells proliferation and sensitizes human primary VS cultures to kinase inhibitors. (A) Cell proliferation in human NF2 schwannoma cells (HEI-193) expressing sh*Ctrl (blue)* or sh*Ncad (red)*, plotted as fold change, ***P < 0.001. (B) Cell viability following treatment with bazedoxifene at the indicated concentrations (nM), normalized to vehicle control (n=3; Bars represent mean ± SD, ****P < 0.0001). (C to L) Cell viability of 5 primary VS cultures (VS102, VS108, VS112, VS119, VS121) treated with either dasatinib (top row) or brigatinib (bottom row), in combination with GC-4 (10μg/mL) or bazedoxifene (BZA, 200 nM). Treatment conditions are indicated beneath each data bar; cell viability is shown as percentage of vehicle control (Bars represent mean ± SD, ns, **P < 0.01, ***P < 0.0001).

We next assessed the therapeutic efficacy of N-cad inhibition in a clinically translatable context using patient-derived primary VS cultures. RNAi-mediated knockdown of N-cad reduced cell proliferation, an effect recapitulated by the pharmacologic N-cad inhibitor BZA (Fig. 5A–B). In five independent primary VS cultures, dasatinib or brigatinib alone led to modest reductions in cell viability. By contrast, co-treatment with BZA or the peptide N-cad antagonist GC-4 consistently enhanced the efficacy of both drugs across four primary cultures (VS102, VS108, VS112, VS119) compared with kinase inhibitor alone (dasatinib, Fig. 5C–G; brigatinib, Fig. 5H–L). VS121 showed limited sensitivity to all treatments likely due to intertumoral heterogeneities in N-cad dependence. Together, these results position N-cad as a context-dependent therapeutic vulnerability in schwannoma cells, supporting a therapeutic strategy by co-targeting N-cad- to enhance the efficacy of kinase inhibitors, particularly in tumors driven by FAK, Src or AKT activation.

### 6. N-cad inhibition enhances brigatinib and dasatinib efficacy in mouse schwannoma models

**Figure 6.**
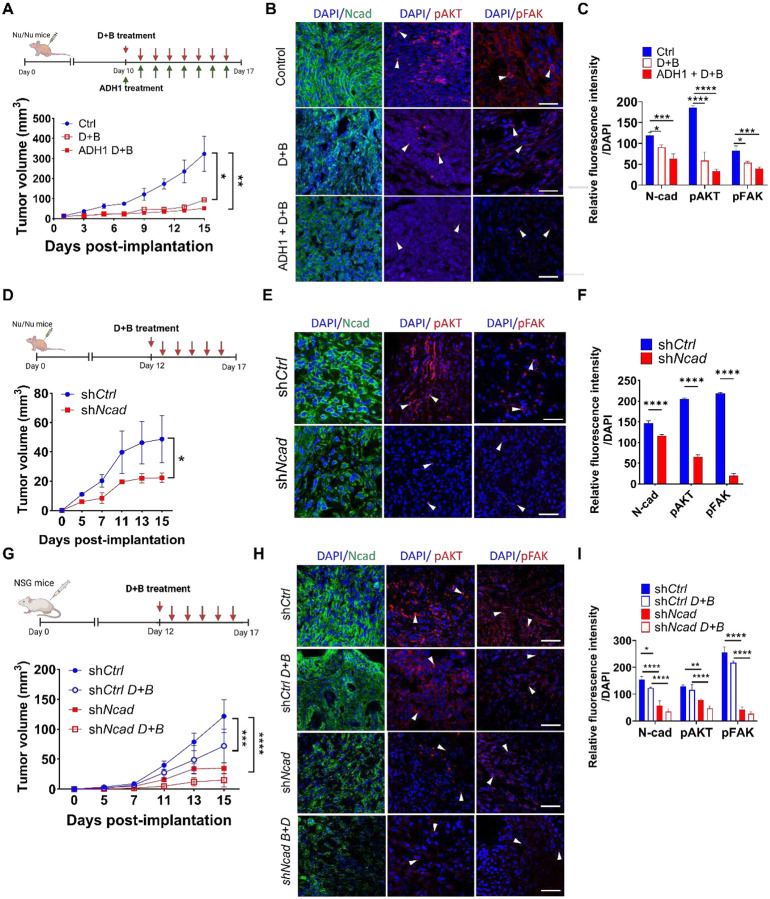
Inhibition of N-cad augments dasatinib + brigatinib activity *and* suppresses AKT/FAK signaling in NF2 mouse schwannoma models *in vivo*. (A) Experimental timeline (top) and tumor growth (bottom) for subcutaneous MD-MSC allografts in nude mice treated with saline (Control), dasatinib (15mg/kg) and brigatinib (25mg/kg) (D+B), or ADH-1 (50mg/kg) in combination with D+B (ADH-1 + D+B), with dosing schedule as indicated (*P < 0.05, **P < 0.01). (B) Representative immunofluorescence staining of tumors from (A) showing N-cad (green), pAKT (red), and pFAK (red). Arrowheads indicate pAKT and pFAK-expressing schwannoma cells. Scale bar, 50 μm. (C) Quantification of N-cad, pAKT, and pFAK fluorescence intensity normalized to DAPI for groups shown in (B) (Bars represent mean ± SD, *P < 0.05, **P < 0.01, ***P < 0.001, **P < 0.0001). (D) Experimental timeline (top), representative tumor images (inset), and tumor growth curves for subcutaneous xenografts of either HEI-193-sh*Ctrl* (blue) or HEI-193-sh*NCad* cells (red) in nude mice treated with D+B. Tumor volume is plotted in mm^3^, (*P < 0.05). (E) Representative immunofluorescence staining of tumors from (D) showing N-cad (green), pAKT (red), and pFAK (red) intensities with DAPI counterstain. Arrowheads indicate pAKT and pFAK positive schwannoma cells. Scale bar, 50 μm. (F) Quantification of N-cad, pAKT, and pFAK fluorescence intensity normalized to DAPI from sh*Ctrl* tumors (blue) and sh*NCad* tumors (red) (Bars represent mean ± SD, ****P < 0.0001). (G) Experimental timeline (top), representative tumor images (inset), and tumor growth curves for subcutaneous xenografts of HEI-193 cells expressing sh*Ctrl* (blue) or shN*Cad* (red) in NSG mice treated with vehicle or D+B (***P < 0.001, ****P < 0.0001). (H) Representative IF staining of N-cad (green), pAKT (red), pFAK (red) and DAPI (blue) in tumors from (G). Arrowheads indicate pAKT, pFAK positive schwannoma cell. Scale bar, 50 μm. (I) Quantification of N-cad, pAKT, and pFAK fluorescence intensities normalized to DAPI for sh*Ctrl* or sh*NCad* tumors treated with saline or D+B (Bars represent mean ± SD, *P < 0.05).

Having established that N-cad inhibition potentiates the anti-proliferative effects of brigatinib and dasatinib *in vitro*, we next asked whether this synergy translates *in vivo* using a mouse schwannoma allograft model (Fig. 6A). MD-MSCs were implanted as subcutaneous allografts in nude mice and treated with dasatinib (15 mg/kg) and brigatinib (25 mg/kg/d, D+B), D+B combined with the ADH1 (50 mg/kg/d), or saline control. By day 15, tumors in untreated mice expanded by 19-fold, whereas D+B limited growth to 7-fold; and adding ADH-1 further constrained tumor expansion to 4-fold (Fig. 6A). Consistent with on-target pathway inhibition, immunofluorescence staining of harvested tumors shows a significant reduction in N-cad, pAKT and pFAK expression, with the strongest suppression observed in the ADH-1 combined with D+B cohort (Fig. 6B–C).

To further validate how N-cad modulates therapeutic response *in vivo*, we established subcutaneous HEI-193 schwannoma xenografts in nude mice using cells expressing either sh*Ctrl* or sh*Ncad*. Compared to xenografts harboring sh*Ctrl*, sh*NCad* tumors showed a significant reduction in tumor burden and a >5-fold decrease of pAKT and pFAK levels in the tumor parenchyma by day 15 (Fig. 6D–E), further confirming that N-cad- associated signaling was indeed suppressed in vivo. Similar results were observed in NSG mice bearing schwannoma xenografts, where N-cad depletion by sh*Ncad* and kinase inhibition suppressed tumor growth, and combined treatment with sh*Ncad* and dual kinase inhibitors (D+B) demonstrated the strongest therapeutic effect, yielding an 8-fold reduction in tumor burden and a significant downregulation of N-cad, pAKT and pFAK expression in tumor tissue (Fig. 6G–I). Collectively, these data identify N-cad as a central mediator that integrates adhesion-dependent FAK and receptor tyrosine kinase-driven AKT/Src signaling. Inhibiting N-cad potentiates TKI antitumor efficacy, providing strong translational rationale for targeting N-cad in combination with kinase inhibition.

## Discussion

Our findings establish N-cad as a novel and key regulator of schwannoma migration, growth and therapeutic resistance. Single-cell and transcriptomic profiling localize N-cad expression to an injury-like non-myelinating Schwann cell population. Clinically, elevated N-cad expression is associated with adverse features including adherent tumor growth and poor hearing outcomes. Elevated N-cad expression favored cohesive, astrocyte-guided schwannoma cells collective migration, whereas reducing N-cad shifted cells toward a more ECM-permissive, dispersive migratory phenotype. N-cad was enriched at the tumor invasive front and leader–follower interfaces and spatially overlapped with MMP-9, consistent with coordinated adhesion remodeling and pericellular proteolysis. N-cad amplified IL-6/STAT3, NF-κB, FAK, Src and PI3K/AKT signaling pathways. Finally, N-cad depletion sensitized primary VS cells and mouse schwannoma to dasatinib and brigatinib.

Surgery remains central to the management of NF2-SWN, yet post-operative morbidities – including profound sensorineural hearing loss, vertigo and facial paralysis – remain substantial [58–60]. Tumors that are large, rapidly growing, or adherent to brainstem and cranial nerves pose additional significant surgical challenges, often require subtotal tumor resection to preserve neurologic function, increasing the risk of tumor recurrence and need for additional surgery [61, 62]. Despite these limitations, no FDA-approved medical therapies exist for inherited or sporadic VS. Targeted therapies against mTOR, VEGF, FAK and PI3K pathways demonstrated limited efficacy and dose-limiting toxicities that preclude long-term use [57, 63, 64]. Bevacizumab, the most effective therapy to date, achieves radiographic or hearing responses in fewer than 40% of treated patients [5]. These limitations highlight the critical need for mechanistically targeted therapies for NF2-SWN patients, ultimately shift away from high-risk surgical interventions.

We demonstrate that schwannoma cells adopted a dynamic leader-follower architecture, where leader cells showed enrichment of N-cad at polarized, filamentous contacts and within Rab5/Rab11-associated recycling endosomes, whereas follower cells maintained a more continuous, junctional N-cad that preserved cohesion during movement, consistent with a dynamic, leader-follower interchange seen in migrating gliomas [39]. Importantly, this leader-follower role is interchangeable and matrix-dependent: in breast cancer, dense collagen increases dynamic remodeling at the leading edge and promotes turnover of leader cells [65]. Furthermore, the colocalization of N-cad with MMP-9 further indicates that N-cad-mediated adhesion is closely coordinated with pericellular proteolysis during migration [66].

Beyond migration, N-cad activates IL-6/STAT3 and NF-kB signaling, promoting a pro-inflammatory niche that enhances schwannoma growth, a mechanism shared across multiple cancers. In breast cancer, N-cad engagement triggers IL-6 and STAT3 activation, with Rac/Cdc42-dependent transactivation of NF-κB positioned upstream of IL-6-gp130/JAK signaling [67, 68]. In glioblastoma, NF-κB-driven IL-6 sustains STAT3 activity and stabilizes stress-adapted tumor states [69]. Homophilic N-cad contacts and elevated surface/recycling N-cadherin regulates coordinated glioma migration on neuronal/astrocytic substrates and limits invasion into ECM-rich matrices [39], underscoring a conserved adhesion-to-inflammation link in tumor progression [70]. Our findings reveal that dysregulated, N-cad-dependent migration along neural interfaces underline adherent growth patterns of aggressive schwannomas.

MMPs, particularly MMP-9, shape tumor behavior by remodeling the ECM and activating matrix-bound factors and cell surface proteins [71]. Building on our previous observation that elevated MMP-9 was associated with adherent schwannomas [10], we show that MMP-9 directly cleaves N-cad into discrete fragments [72]. MMP-9 also directly amplifies a pro-inflammatory milieu by releasing cytokines and activating NF-kB and IL-6/STAT3 [73], indicating that MMP-9 overexpression could further drive N-cad-dependent tumor growth.

N-cad function is further influenced by catenin-mediated stabilization and trafficking, with p120-catenin limiting N-cad endocytosis and β-/α-catenin coupling cadherins to the actin cytoskeleton. This layer is particularly relevant in NF2-schwannomas, where merlin loss results in immature, unstable adherent junctions and disrupts cadherin-β-catenin organization, reducing the threshold for junctional disassembly during tumor invasion [74–77]. Although catenins were not directly examined, prior studies linked β- and p120-catenin to N-cad stabilization and IL-6/STAT3 activation [78]. Together, these observations establish a targetable MMP-9/N-cad/catenin axis, where MMP-9–driven N-cad cleavage and impaired junctional stability promote leader migration and engages IL-6/STAT3 and NF-κB signaling during schwannoma progression.

Kinase inhibition has shown limited and variable efficacy in NF2-SWN clinical trials, consistent with adaptive survival signaling in merlin-deficient schwannoma cells. Here, N-cad inhibition reduced FAK, SRC and AKT activity, lowering the signaling “set point” to sensitize tumors to dasatinib and brigatinib, consistent with prior evidence that compensatory FAK activation limited responses to PI3K inhibition in NF2 schwannoma and co-targeting PI3K and FAK was more effective than either alone [64]. Despite encouraging preclinical data on multi-kinase inhibition strategies [56, 57] and clinical activity of brigatinib in a recent pivotal trial for NF2-SWN, durable tumor control rates remained low [7, 55, 79]. Our data supports N-cad co-inhibition as a dose-sparing combination strategy; clinical trials using ADH-1 (Exherin) showed acceptable safety but limited efficacy, supporting a dose-sparing combination strategy rather than monotherapy [80]. More broadly, these findings support strategies moving beyond “kinase-on-kinase” inhibition. Our findings identify N-cad as a convergent hub linking FAK/SRC and PI3K/AKT pathways, whose disruption weakens the adhesion-dependent axis and exposes a therapeutic vulnerability that sensitizes schwannomas to kinase inhibitors and PI3K-mTOR-directed agents.

This study has several limitations. First, the patient-derived cohort was modest in size, and inter-tumoral heterogeneity likely contributed to variable baseline phenotypes and therapeutic responses to N-cad inhibition; this can be addressed by expanding clinically annotated cohorts and stratifying analyses by tumor subtype and clinical features in the future. Second, while our cell-based assays and xenograft/allograft models enable mechanistic and pharmacologic testing, they do not fully recapitulate the native IAC-CPA microenvironment, including immune/stromal context that may shape tumor adhesion and invasion. An orthotopic tumor model in the CPA/IAC using immunocompetent or humanized mouse models will enable assessment of immune cell and stromal interactions. More sophisticated organotypic/co-culture platforms can be developed to mimic interactions between schwannoma cells and ECM. Third, although ADH-1, GC-4, and BZA support convergent targeting of N-cad, we did not comprehensively quantify *in vivo* pharmacokinetics (PK), pharmacodynamics (PD) or target engagement, and off-target effects cannot be fully excluded. Future studies will evaluate PK/PD and drugs response through orthogonal genetic approaches, using Cre/lox-based conditional N-cad knockout systems. Fourth, our imaging suggests enhanced N-cad trafficking in leader-like cells, but the causal roles of specific endocytic/recycling routes and catenin partners remain to be resolved. This can be dissected by live cell time lapse imaging of fluorescently tagged N-cad combined with antibody-based surface pulse-chase/internalization-recycling assays, alongside genetic or pharmacologic perturbation of endocytic/recycling regulators and catenin binding. Finally, while we demonstrate MMP-9 dependent N-cad cleavage, the functional consequences of the resulting fragments on cohesion, migration, and signaling will require dedicated gain-/loss-of-function studies.

## Conclusion

Our findings identify N-cad as a novel and key regulator of schwannomas migration, proliferation, and therapeutic vulnerability. Through both genetic and pharmacologic approaches, we demonstrate that N-cad promotes schwannoma progression by modulating cell–cell adhesion and activating pro-tumorigenic IL-6/STAT3, NF-κB, and FAK/AKT signaling. Targeting N-cad not only impairs these oncogenic pathways but also reveals a therapeutic vulnerability that enhances the efficacy of dasatinib and brigatinib. These results provide a strong preclinical rationale for incorporating N-cad–directed strategies into future therapeutic approaches for NF2-SWN.

## Supplementary Material

Supplementary Files

This is a list of supplementary files associated with this preprint. Click to download.

- Supplementaryinformation.pdf

## Data Availability

The data will be made available upon request. Please reach out to the corresponding author (Y.R., H.T.N.)
